# Host Plant Constancy in Ovipositing *Manduca sexta*

**DOI:** 10.1007/s10886-021-01309-3

**Published:** 2021-09-21

**Authors:** Nandita Nataraj, Elisabeth Adam, Bill S. Hansson, Markus Knaden

**Affiliations:** Department of Evolutionary Neuroethology, Max Planck Institute for Chemical Ecology, 07745 Jena, Germany

**Keywords:** Moth, Behavior, Host plant, Oviposition, *Nicotiana attenuata*, *Datura wrightii*

## Abstract

Many pollinating insects exhibit flower constancy, i.e. they target flower species they have already experienced and fed from. While the insects might profit from reduced handling costs when revisiting similar flowers, flower constancy, in addition, is of benefit for the plants as it guarantees pollen transfer to conspecifics. Here we investigate whether the previous experience of an insect can also result in oviposition constancy, i.e. whether ovipositing on a given plant species will drive future oviposition preference in a female insect. We show that female hawkmoths (*Manduca sexta*), after having oviposited on a given plant species only once, indeed will prefer this plant in future oviposition choices. As oviposition preference is even affected 24 h after the moth has oviposited on a given plant, long term memory seems to be involved in this oviposition constancy. Our data furthermore suggest that, as shown for flower constancy, ovipositing moths increase their handling efficiency by targeting those host plants they have already experienced.

## Introduction

Pollinators play an important role in maintaining the terrestrial ecosystem with more than 80% of flowering plant species relying on insect pollination (Ollerton et al. 2011). At the same time, herbivore insects and/or their offspring can cause huge damage to their host plants (Delaney et al. 2001). Therefore, plants should advertise themselves to potential insect pollinators, while hiding from insect herbivores. The relationship of the tobacco hawkmoth *Manduca sexta* and its host plants *Datura wrightii* and *Nicotiana attenuata* is specifically interesting as the moth is one of the most important pollinators of these plants, while its caterpillars can cause severe damage both on *Datura* and *Nicotiana* (Zhang et al. 2019).

The mutualistic relationship between plants and their pollinators often has resulted in a so-called flower constancy, where pollinators like bees (Gruter and Ratnieks 2011) or butterflies (Goulson et al. 1997) learn features of flowers they have successfully foraged from and afterward specifically target similar flowers during their foraging flights. While the insect might profit from reduced handling costs when revisiting similar flowers, the plants profit from the guaranteed pollen transfer to conspecifics (Waser 1986).

Foraging tobacco hawkmoths exhibit an innate preference for specific flowers (Haverkamp et al. 2016) and floral odors (Bisch-Knaden et al. 2018). Such preferences for certain volatiles potentially enable pollinators to rapidly detect valuable floral resources without prior sampling. However, pollinators also show accurate learning abilities for those compounds reliably associated with a flower reward (Knauer and Schiestl 2015). This behavior might enable pollinators to switch between flower species, as soon as the most preferred flowers are not available anymore and are a prerequisite for the above mentioned flower constancy.

Indeed, also the tobacco hawkmoths’ flower preference can be altered by learning. Having successfully foraged from a flower only once, the moths will target the flower’s odor plume again, even if it was not innately attractive to the insect (Cook et al. 2020; Riffell et al. 2008).

Here we ask, whether ovipositing hawkmoths exhibit an innate preference for one of their host plants, *Datura* or *Nicotiana*, and whether previous oviposition experience with one of these plants results in a changed preference. We thus investigate, whether the moths exhibit any kind of learned oviposition constancy.

We find, that naïve moths slightly prefer to oviposit on *Datura* rather than on *Nicotiana*, when they are offered individual leaves of both plants. However, we furthermore show, that a single oviposition experience with either *Datura* or *Nicotiana* is sufficient to strongly tune the moths’ preference towards the same plant, and at least for *Datura* moths can remember this experience even 24 h later.

To our knowledge, a similarly fast and long lasting learning of oviposition cues so far has been reported in parasitoid wasps only (Smid et al. 2007).

## Methods and Materials

Insect rearing: All animals originate from eggs collected in Utah (DI Ranch, Santa Clara, UT, USA) in 2009 and since then were reared at the Max Planck Institute for Chemical Ecology, Jena, Germany. Female moths were allowed to oviposit on *Datura* plants. Eggs were collected immediately after oviposition and larvae were subsequently maintained on an artificial diet at 40% relative humidity and 26 °C with a light: dark cycle of 15:9. Fifth-instar caterpillars were individualized for pupation and left till 1 week before adults eclosed from the pupae at the same climate conditions. Pupae were sexed, and male and female pupae were transferred to separated flight cages with a light: dark cycle of 16.5:7.5, 60% relative humidity, and 25 °C during the light phase, and 60% relative humidity and 20 °C during the dark phase. Two-day-old female and male hawkmoths were kept for mating for 24 h. After roughly 6 h moths that did not mate were removed from the cage and only pairs that were mating were kept. The mating cage was devoid of any plant material and sugar water. During mating, the male: female ratio was 1:1 or 2:1 depending on the availability of adult moths. Only mated female moths were used for the oviposition experiments.

Plant Growth: The plant samples were grown in the greenhouse of the Max-Planck Institute. Wild type *Datura* and *Nicotiana* seeds were collected from the desert in Utah and then over generations were grown in greenhouse culture under 16 h of light, with temperature during day time set at 23–25° Celsius and at night 19–23° Celsius, humidity during the entire day was set to 50–60 percent. The seeds were initially potted in a germination tray. After 20 days the saplings were transferred to 1 L pots. Once the plants reached a height of roughly 50 cm (*Nicotiana*: 80 cm) they were used for the experiments. At least 1 week before the experiment plants were transferred to a climate chamber with the same settings as the moth flight cage.

Behavioral wind tunnel assay: In a wind tunnel (plexiglass, 250 cm × 94 cm × 90 cm; airflow, 0.4 m/s; light, photosynthetic active radiation: 0.27 μmol/[m^2^s]; temperature, 25 °C; humidity, 70%) female hawkmoths were tested for their oviposition preference for *Datura wrightii* and *Nicotiana attenuata*. For every trial, a freshly excised leaf and a fresh moth were used. We selected leaves to match the surface area of the *Datura* and *Nicotiana* leaves in the choice assay.

Single leaves of both plants were placed on pillars (height of the pillar, 40 cm; diameter of each pole, 2 cm; distance between pillars, 45 cm) at the upwind end of the wind tunnel. Individual moths were kept in circular cages (diameter, 13 cm; height, 15 cm) in a pre-exposure chamber at the same temperature, humidity, and light as in the wind tunnel for about 1 h before the experiment.

For testing, the opened cages were placed on a take-off platform positioned at the downwind side within the wind tunnel. Each moth was given 2 min to initiate wing fanning (which usually precedes the moths’ take-off) to be considered for analysis. After taking off, moths were recorded with five Logitech cameras (spatial resolution, 1280 × 720 pixel; temporal resolution, 30 frames per second).

**Fig. 1:**
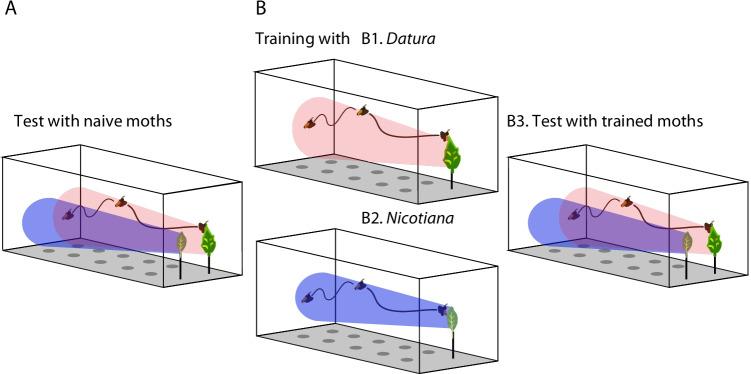
Experimental paradigm to test for any effect of experience on the moths’ oviposition preference. **A** Innate preference; Test for the moths’ innate oviposition preference for single leaves of *Datura* and *Nicotiana*. **B** Learned preference; Moths are allowed to oviposit once on either *Datura* (**B1**) or *Nicotiana* (**B2**) and their preference for single leaves of *Datura* and *Nicotiana* becomes tested afterward (**B3**)

We asked the following four questions:

1. Do naïve ovipositing hawkmoths exhibit an innate preference for one of the host plants *Nicotiana attenuata* or *Datura wrightii* over the other?
  Naive moths were exposed in the wind tunnel to two individual leaves of *Datura wrightii* and *Nicotiana attennuata*.
  During five minutes of flight, the first choice and the total number of eggs laid on each leaf were recorded (Fig. 1A)
2. Would oviposition experience alter a moth’s oviposition preference?
  and, if so,
3. How long would the memory last?

Naïve moths were exposed to a single leaf of either *Datura wrightii* or *Nicotiana attenuata* (Figs. 1B1 and 2) in the wind tunnel*.* As soon as the oviposition started, the animals were captured, resulting usually in a single egg (at max six eggs) laid before the moth was removed from the wind tunnel. These from now on called “experienced moths” were tested for their oviposition preference either 30 min after their oviposition experience, or 1 day later: As before, the now experienced moths were exposed in the wind tunnel to two individual leaves of *Datura wrightii* and *Nicotiana attenuata*. Again, during five minutes of flight, the first choice and the total number of eggs laid on each leaf were recorded (Fig. 1B3).

4. Does experience increase oviposition efficiency?
  As mentioned above, naïve moths were exposed to leaves of either *Datura* or *Nicotiana* in the wind tunnel*,* and the time from starting flight until they laid their first egg was measured. The same – now experienced – moths were 30 min later again exposed to a leaf of the trained plant and the time from starting flight until laying the first egg was measured (experimental set up as in Fig. 1B).
  All data obtained were first checked for normal distribution. Since data were not normally distributed we performed non-parametric tests for statistical analysis. Details of statistics are provided in figure legends.

## Results

**Fig. 2:**
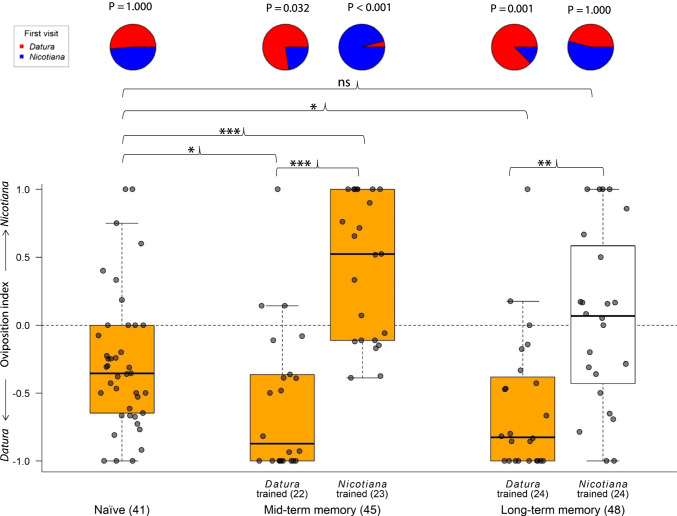
*Manduca sexta*’s ovipositing preference can be altered by experience. First choices (pie charts) and oviposition indices (boxplot diagrams) of naïve and experienced *Manduca* females. Left panel, naïve moths; middle panel, experienced moths tested 30 min after experiencing either *Datura* or *Nicotiana*; right panel, experienced moths tested 1 day after experiencing either *Datura* or *Nicotiana*; sample sizes for each group are given in brackets*.* Oviposition index for each moth was calculated as (number of eggs on *Nicotiana*—number of eggs on *Datura*)/total number of eggs. First choice: Pie charts, p-values below 0.05 depict significant preference during the first choice. Oviposition index: colored box plots depict an index different from 0 (p < 0.05, Wilcoxon signed rank test). Boxplots give the median (black line), quartiles (box), and 95% confidence intervals (whiskers). Brackets between boxplots depict differences between treatment groups (Kruskal Wallis analysis with selected pairwise Wilcoxon rank sum test corrected for repeated analyses)

Moths exhibit an innate preference for *Datura*: To test whether gravid females exhibit an innate preference, we offered naïve females single leaves of *Datura* and *Nicotiana* in a 2-choice assay in a wind tunnel. While the moths contacted both leaves equally often during the first leaf investigation (Naïve; Fig. 2), the females laid more eggs on *Datura* than on *Nicotiana* leaves during the 5 min of observation (Naïve; Fig. 2). We conclude that visual and olfactory cues of *Datura* and *Nicotiana* are equally strong to attract naïve females over distance, but that, when it comes to oviposition, *Datura* leaves are more attractive to the moths than those of *Nicotiana*.

Learning alters oviposition preference: When we tested experienced moths, i.e. moths that had already oviposited either on a *Datura* or *Nicotiana* leaf, we found altered preferences when compared to naïve moths. Moths that were tested 30 min after they had experienced a leaf of a given plant species, exhibited a strong preference for this species regarding their first choice (Mid-term memory; Fig. 2). In addition, moths that experienced *Datura*, exhibited an even increased oviposition preference for *Datura* as compared to naïve moths. Moths that, however, experienced the innately less preferred *Nicotiana* later reversed their oviposition preference towards this plant (Mid-term memory; Fig. 2). While the increased preference for *Datura* after experiencing it once remained even after 24 h, the learned preference for *Nicotiana* disappeared after this time period (Long-term memory; Fig. 2).

**Fig. 3:**
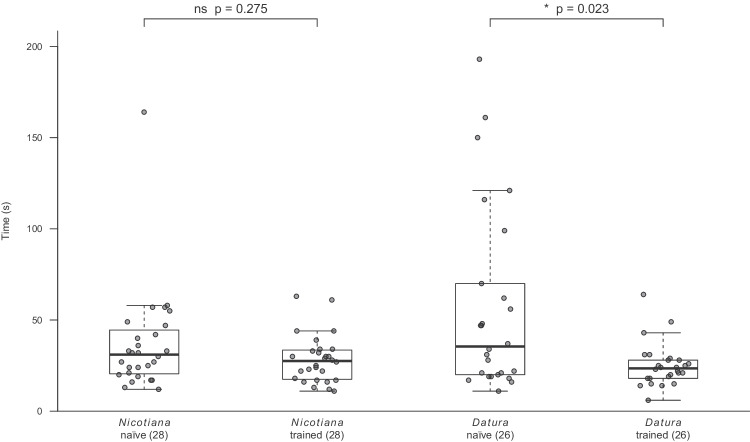
Experience can increase oviposition efficiency. Time from starting flight until oviposition in naïve and experienced moths. Boxplots give the median (black line), quartiles (box), and 95% confidence intervals (whiskers). Square brackets, p-values below 0.05 depict significant differences between naïve and experienced moths (Wilcoxon matched-pairs rank sum test). The sample size for each group is given in brackets

In order to test, whether the experience does not only change the moths’ preferences but also increases their oviposition efficiency, we tested, whether experienced moths would approach a given leaf and oviposit on it faster than naïve moths. While experience with *Nicotiana* did not result in any increased oviposition efficiency, moths that had oviposited on a *Datura* leaf once, later took significantly less time to approach the leaf and oviposit on it (Fig. 3).

## Discussion

Our findings show that learning alters the oviposition preference in mated female *Manduca sexta* moths. Naïve moths, when exposed to individual leaves of *Datura wrightii* and *Nicotiana attenuata*, do not exhibit any preference regarding their first choice but finally lay more eggs on the *Datura* leaf. The latter is in agreement with another study, where hawkmoths, when tested with whole plants of four species including *Datura* and *Nicotiana*, deposited most of their eggs on the *Datura* plants (Späthe et al. 2013b). This oviposition preference might be due to visual and/or olfactory cues. However, it is of course also possible that the moth’s final decision to lay an egg is driven by tactile, i.e. gustatory or physical, cues after the moth has contacted the leaf. In field experiments, *Manduca sexta* females exhibited an equally strong preference to oviposit on *Datura* and *Nicotiana* plants as compared to other potential host plants (Garvey et al. 2020). It is usually assumed that female moths prefer host plants on which their offspring performs best. Larval performance depends both on the nutritional value of the plant and on potential protection the larvae get from the plant against predators and parasitoids. *Manduca sexta* was e.g. shown to prefer *Nicotiana* over *Solanum* plants, although their larvae grow much faster on *Solanum.* The reason for that might be the significantly lower parasitization rate of *Manduca* larvae when they grew on *Nicotiana* plants (Garvey et al. 2020). The reason for the innate preference for *Datura* described in our study remains open. In our lab colony, we provide females always with *Datura* plants for oviposition. By that, we might have selected for a *Datura* preference in the breeding situation*.*

However, after having a single chance to oviposit on a given leaf of only one of the two plants, and being then offered leaves of both species again, females immediately choose the leaf of the plant they have experienced already, and during 5 min of flight deposit a significantly higher number of eggs on this leaf (Fig. 2). It seems that the oviposition experience drives the moth’s preference during consecutive oviposition events. We also show, that a single oviposition experience is enough to affect the moth’s preference even after 24 h (Long-term memory; Fig. 2). Interestingly, this holds true for the experience with *Datura* only, where the first choice of the moths was still significantly turned towards this plant 24 h after ovipositing on it. Obviously, the learning of the innately less preferred *Nicotiana* plant is weaker, as the strong preference that we observed 30 min after the experience with *Nicotiana* was completely abolished after 24 h. It remains open, whether the moths’ memory for *Datura* lasts longer, because of any potential selection for *Datura* preference during the breeding process (*Datura* is provided for oviposition in our breeding colony). Future studies shall reveal, whether maybe even just the exposure to a host plant odor without any oviposition experiences can affect the moths’ later preference and whether repeated oviposition on a given plant species even increases the memory duration.

To our knowledge, a comparatively fast learning of oviposition cues so far has been shown only in parasitoid wasps (Kaiser et al. 2003). When the wasp *Leptopilina boulardi* oviposits in a *Drosophila* larva while being exposed to a specific odorant, it associates this odorant with the host, and will later target this olfactory cue again. While *L. boulardi* needs several training trials to form a long-term memory, the close relative *L. glomerata*, requires only one trial to still remember the odorant after one day (Smid et al. 2007). With keeping their increased preference for *Datura* despite a single oviposition experience even after 24 h, *Manduca sexta*’ learning capabilities seem to match those of *L. glomerata*.

The formation of an associative memory between a given odor and food has been investigated in many other insects including (among others) ants (Huber and Knaden 2018), wasps (El-Sayed et al. 2018), and even mosquitos (Tomberlin et al. 2006)*.* Also, *M. sexta* has been shown to increase its preference for the odor of innately less attractive flowers after feeding on them (Cook et al. 2020; Riffell et al. 2008). Learning of food odors can thus result in food constancy, where the insect focuses its foraging behavior on food sources it had good experience with.

Our knowledge regarding such constancy behavior in herbivorous insects is mainly limited to flower constancy. Here, insect pollinators tend to restrict their visits to those flower types they have already successfully fed from, and at the same time ignore potentially rewarding flowers they have no experience of. Different explanations of why flower constancy could be adaptive for the insect have been well reviewed (Amaya-Márquez 2009). One possible explanation is that insects can handle only limited information at a time, such as suggested by the ‘short memory limitation theory’. Another possible explanation comes from the ‘learning investment hypothesis’ (Chittka et al. 1997, 1999; Menzel et al. 1998; Raine and Chittka 2005), which predicts that the investment required by an insect to learn how to handle a new flower type is higher than the possible gain coming from a switch in flower type. Overall, switching between flowers with different morphologies can increase total flower handling time. Therefore, foraging insects might remain flower-constant to optimize their costs per benefit (Chittka et al. 1997; Laverty 1994).

While learned flower constancy is well investigated, less is known about learned oviposition constancy as we describe in the present study. Finding a suitable host plant for oviposition is crucial for the fitness of herbivorous insects as it affects the survival of their offspring. It was shown before that the generalist *Spodoptera littoralis* preferentially oviposits on host plants it fed on during its larval stage (Lhomme et al. 2020). Obviously, upon feeding on a given host plant, the larva learns host-specific cues, which drive its oviposition behavior when it becomes an adult. In this case, larval feeding can be interpreted as the rewarding act that leads to a shift in host-plant preference. It was also shown, that moths that as adults experienced a host plant for several days, later preferred this species over other host plant species they had not experienced before (Cunningham et al. 1998). In our case, however, the adult hawkmoth changes its preference after a single oviposition event. To oviposit on a given host plant, the moth first follows the plume of the plant, targets the leaf by both vision and olfaction, then probes it with the legs and the ovipositor which might provide both gustatory as well as mechanosensory information, before it finally decides whether or not to lay an egg. We show that the decision to lay an egg results in a strong preference to target the same host plant species again. As we performed all experiments with real leaves, we cannot tell, whether the learned preference is mainly driven by visual or olfactory cues. Further investigations will also reveal which part of the oviposition process is rewarding to the moth and finally leads to the shift in preference. Is it the process of the actual egg delivery or is any kind of sensory feedback the moth experiences upon first leaf investigation sufficient to later make the moth target the same plant again? And what are the cues that dictate, whether the moth still remembers one plant after 24 h (*Datura*), but forgets about the other (*Nicotiana*)?

Despite these open questions, we can still answer, whether the moth potentially profits from its learned oviposition constancy. When experiencing a leaf of a given plant once, the female moth probably does not learn too much about, whether this plant is good or not for the performance of the moth’s offspring. This knowledge should be rather inherited and should govern the innate oviposition preference of the female moth. Any experience the moth gets during oviposition should rather affect the female’s own future performance. In the wind tunnel naïve moths took more time to target a *Datura* leaf as compared to a *Nicotiana* leaf. This might be due to the comparatively low amount of volatiles released by *Datura* (Späthe et al. 2013a). However, animals that had experienced *Datura* already, targeted the olfactory inconspicuous *Datura* leaves significantly faster than naïve animals (Fig. 3). It could be that experience led to the formation of a search image – as also discussed for bees when establishing flower constancy (Amaya-Márquez 2009). Since flight for a gravid female is costly, both in terms of energy as well as due to potential predators, learning that increases the efficiency in targeting host plants could be seen as adaptive. An increasing oviposition efficiency due to experience has also been observed in three species of *Colias* butterflies. Females of these species lay single eggs on legume plants and when searching for further hosts become more and more efficient in discriminating legume plants from visually similar non-host plants (Stanton 1984). A similarly increased efficiency to locate a host plant after oviposition experience with this plant was also shown in two other generalist butterflies (*Polygonia c-album* and *Vanessa cardui*). Both became faster in localizing a given host among non-host plants after they had gained oviposition experience on it (Gamberale-Stille et al. 2019). One should mention, that the female moths in our experiments experienced isolated leaves only. This is of course an artificial situation, as in the field both *Datura* and *Nicotiana* are visited by the moths for oviposition and nectar feeding. It is furthermore known that nectar amount and nectar chemistry can affect the moths subsequent oviposition behavior on that plant (Adler and Bronstein 2004). While our findings suggest that oviposition experience is sufficient to change the moths’ oviposition preferences, feeding experience at the flowers of those plants may have further effects.

Finally, to survive, insects should be able to learn and adapt to changes in the ecosystem. With varying climatic conditions, host plant species diversity tends to change. Fast flying moths like *Manduca sexta* might during their life therefore experience habitats with different host plant species. A non-changeable preference for a given host plant could hence result in reduced numbers of offspring if a female moth ends up in a habitat that does not provide this specific plant. At the same time, human-derived pollutions like ozone and nitric oxides have been shown to change plant emissions (Farré-Armengol et al. 2016; Fuentes et al. 2016). It was shown that *Manduca sexta* can learn ozone-induced changes in floral odors (Cook et al. 2020), and might therefore still be able to forage despite the degrading effects pollutants have on its innately attractive flower odors. We do not know yet, whether olfactory driven host choice is affected by degrading pollutants also. A flexible host preference, however, that can be changed upon experience might help insects to locate suitable hosts even under these less predictable conditions.

## Data Availability

All data are presented in the manuscript.
